# Laparoscopic versus open pancreatoduodenectomy: a pilot randomized trial in a developing African country

**DOI:** 10.1007/s00464-025-12377-x

**Published:** 2025-12-04

**Authors:** Mohamed Saber Mostafa, Dina Hamour, George Abdelfady Nashed Aiad, Hany Armia Balamoun, Mohamed Hamdy Khattab, Mohamed Nasr Shazly, Mohamed Elshawadfy Nageeb, Abdelkarem Ahmed Abdelkarem Mohamed

**Affiliations:** General Surgery Department, Kasr Al Ainy School of Medicine, Cairo University, Giza, Egypt

**Keywords:** Pancreatoduodenectomy, Laparoscopic approach, Open surgery, Safety, Developing world, Africa

## Abstract

**Background:**

Pancreatoduodenectomy (PD) is a major abdominal surgery. While laparoscopic PD (LPD) is gaining acceptance globally, its feasibility and safety in low-resource settings require further evaluation. This study aimed to identify the benefits and drawbacks of LPD compared to open PD (OPD) in a developing African country.

**Patients and methods:**

This randomized controlled trial included patients with oncologic indications for PD. Participants were randomized into two groups: LPD and OPD. The primary endpoint was length of hospital stay, postoperative morbidity and mortality, blood loss, and the need for transfusion of blood products. The secondary outcomes were the operative time in minutes, pain VAS scores, duration to ambulation, gastrointestinal (GIT) recovery, and the quality of oncological resection.

**Results:**

A total of 68 patients were initially screened for eligibility, of whom 48 were randomized and underwent pancreatoduodenectomy (LPD: *n* = 30; OPD: *n* = 18). Twenty patients discontinued the intervention after randomization due to withdrawal or peri-induction instability, resulting in a dropout rate of 29.4%. The duration of hospital stay was significantly shorter in the LPD group (8.1 ± 5.6 days) than the OPD group (10.6 ± 6.1 days, *p* = 0.049). LPD also demonstrated lower intraoperative blood loss, reduced transfusion rates, faster gastrointestinal recovery, and earlier ambulation. Oncologic outcomes, including margin status and lymph node yield, were comparable between groups.

**Conclusion:**

LPD resulted in a significantly shorter hospital stay compared to OPD, confirming its benefit in postoperative recovery. Despite a longer operative time, LPD showed advantages in blood loss, transfusion needs, and functional recovery, while maintaining comparable oncologic safety. These findings support the feasibility and potential value of LPD in selected patients, even within a resource-constrained setting.

**Supplementary Information:**

The online version contains supplementary material available at 10.1007/s00464-025-12377-x.

Pancreatoduodenectomy (PD) is a complex abdominal surgery involving the resection of the pancreatic head, duodenum, and common bile duct (CBD), with or without partial gastrectomy. Despite advancements in surgical techniques and perioperative care, PD still carries substantial risks of complications and mortality [1]. It remains the cornerstone treatment for malignant tumors of the pancreatic head and periampullary region, and it is also indicated in selected cystic neoplasms, neuroendocrine tumors, and isolated metastases [2, 3].

Pancreaticoduodenectomy was not accepted until Whipple introduced a two-stage PD in 1935 [4]. Whipple then described a successful one-stage PD [5]. The open approach to PD has long been considered the standard. However, minimally invasive approaches have been evolving since Gagner and Pomp first reported LPD in 1994 [6]. Initial experiences were associated with high conversion rates and limited benefits, leading to skepticism [7]. Since then, laparoscopic approaches have been growingly studied, aiming to define the presumed benefits of this minimally invasive approach [8, 9].

Over the past decade, however, several non-randomized and randomized studies—including the LEOPARD-2 trial—have assessed the feasibility, safety, and potential advantages of LPD over open OPD, particularly in terms of recovery time and reduced surgical trauma [10].

Additionally, the robotic-assisted approach to PD has emerged as a promising alternative, offering improved dexterity and precision, especially in complex reconstructive steps. Recent randomized data have started to support the use of robotic PD in specialized centers, although accessibility remains limited by cost and infrastructure [11, 12].

Despite these advances, the implementation of minimally invasive PD—both laparoscopic and robotic—remains uneven across different healthcare systems, particularly in developing countries. This study aimed to evaluate the short-term outcomes and feasibility of LPD compared to OPD in a resource-limited African setting, where surgical innovation faces unique logistical and economic barriers.

## Patients and methods

This was a randomized controlled trial that was performed at the Surgery Department of our institution in a developing African country during the period from September 2022 to December 2023. The study was registered in ClinicalTrials.gov with registration number NCT06661135, https://clinicaltrials.gov/study/NCT06661135?term=NCT06661135&rank=1. The study was approved by the local Research Ethics Committee (REC) with approval number MD-169–2022. The study followed CONSORT 2010 guidelines, including the use of a CONSORT flow diagram and checklist.

### PICOS Statement

- *Population (P):* Adult patients with oncologic indications for pancreatoduodenectomy (PD) admitted to our institution.
- *Intervention (I):* Laparoscopic pancreatoduodenectomy (LPD).
- *Comparison (C):* Open pancreatoduodenectomy (OPD).
- *Outcomes (O):* Primary outcomes were ICU and overall hospital stay duration (recorded in days), postoperative morbidity (Clavien–Dindo classification [13]) including postoperative pancreatic fistula (POPF) (was defined according to the 2016 update of the International Study Group of Pancreatic Surgery (ISGPS) [14]), 30-day mortality (defined as death occurring within 30 days of surgery), intraoperative blood loss (was defined as the volume in milliliters collected in suction canisters minus irrigation fluid, plus the weight of sponges), and transfusion needs (recorded as number of patients required transfusion during or after surgery. The need for transfusion was defined as administration of any packed red blood cells during or within 24 h after surgery). Secondary outcomes included operative time (measured in minutes from skin incision to closure), pain scores (assessed using the Visual Analog Scale (VAS) ranging from 0 (no pain) to 10 (worst pain) [15]), ambulation time (the number of hours or days until the patient could walk independently), gastrointestinal (GIT) recovery (time to first flatus and bowel movement), oncological resection quality, assessed by margin status (R0, R1) and lymph node yield, according to pathology reports. Composite outcomes included Textbook Outcome (TO: A composite measure reflecting an "ideal" postoperative course. It is defined as the absence of major complications [Clavien–Dindo grade ≥ III], no CR-POPF, no PPH, no reoperation, and no mortality [16]), Failure to Rescue (FTR: Death occurring in patients who experience a major complication [Clavien–Dindo grade ≥ III] [17]), and PAncreatic Surgery Composite Endpoint (PACE: A composite endpoint that includes clinically relevant POPF, PPH, DGE, bile leak, reoperation, and mortality [18]). This composite measure provides a comprehensive assessment of postoperative outcomes in pancreatic surgery.
- *Study Design (S):* Prospective, randomized controlled trial (RCT).

### Inclusion criteria

- Adult patients (≥ 18 years old) admitted with oncological indications for PD.
- Patients deemed operable based on preoperative work-up.
- Patients who provided informed consent.

### Exclusion criteria

- Patients found inoperable during preoperative work-up.
- Patients with large ventral hernias.
- Patients with irresectable tumors (e.g., arterial encasement > 180°, unreconstructable SMV/PV occlusion, or distant metastasis).
- Patients unfit for laparoscopic surgery due to contraindications.

Patients with borderline resectable or locally advanced tumors (patients with limited venous involvement amenable to reconstruction or ≤ 180° arterial abutment without encasement) were evaluated on a case-by-case basis and included only if preoperative imaging confirmed resectability.

The initially included patients were subjected to a dedicated clinical examination by a multidisciplinary team, including history taking, physical assessment, routine laboratory work-up, including analysis of carbohydrate antigen 19–9 (CA 19–9), as a tumor marker, and radiological assessment. Threshold levels for elevated CA-19–9 were considered at > 37 U/mL [19]. Multi-phase pancreatic protocol pelvi-abdominal computed tomography (CT) examination with IV contrast was performed to assess neoplastic mass characteristics, extension, vascular invasion, lymph node involvement, and potential metastatic lesions. The scan was performed with a slice thickness of 1–2 mm to achieve high-resolution images. Endoscopic ultrasound (EUS) was done for all patients using an EG-3870UTK attached to a Hitachi Avius sonographic machine under deep sedation. Preoperative drainage was done for patients whose serum bilirubin levels exceeded 12 mg/dL and those with debilitating pruritus or cholangitis.

### Sample size calculation

The sample size was assessed using the G ∗ power 3.1.9.4 software (Universities Kiel, Germany). The calculation was based on the study of Ammori et al. [20], which reported a mean hospital stay length of 4.7 ± 1.3 days in the OPD group compared to a respective mean of 8.9 ± 5.4 days in the LPD group. However, to improve the power and validity of analyses (especially for complications), we increased the enrollment to 34 per group prospectively, before unblinding. We prospectively inflated the target sample from 12 to 34 to safeguard power if late exclusions occurred, as we anticipated, based on our experience in this clinical setting, that a significant number of patients might later withdraw consent, become ineligible due to intraoperative findings, or face logistical and financial constraints leading to dropouts. Our setting amplifies this risk: (i) many families exercise a culturally sanctioned “last-minute veto” once the magnitude of surgery becomes tangible; (ii) intraoperative cancelation is frequent in a low-resource ICU where any accidental event may mandate postponement; and (iii) during protocol drafting (mid-2022), sporadic COVID-19 surges were still triggering routine PCR screening and compulsory isolation. Without this planned over-recruitment, our analyzable cohort would have fallen below the minimum required, and the study would have been underpowered despite full adherence to CONSORT principles.

### Randomization

The enrolled patients were randomly assigned in a 1:1 ratio to one of two intervention groups: the OPD group, which underwent the conventional open surgical procedure, or the LPD group, which underwent the minimally invasive laparoscopic approach. Randomization was performed using a simple randomization method with sealed, opaque, and sequentially numbered envelopes to ensure allocation concealment. These envelopes contained group assignments and were prepared by an independent researcher not involved in patient recruitment, assessment, or treatment. Upon enrollment and after obtaining informed consent, each patient was asked to pick one envelope from the shuffled set in the presence of the independent colleague. The selected envelope was then opened to determine the patient's group assignment. This process ensured that both investigators and participants were unaware of group allocation prior to randomization, thereby minimizing selection bias. Blinding of outcome assessors was also implemented to minimize detection bias, although due to the nature of the intervention, blinding of participants and care providers was not feasible. Standardized protocols were applied across all study sites to ensure consistency in intervention delivery and outcome measurement. Data analysts were also blinded to group allocation during statistical analyses to further reduce interpretation bias.

### Surgical intervention

In the OPD group, a bilateral subcostal incision was used. In the LPD group, induction of pneumoperitoneum then trocar insertion, about 5 or 6 trocars were inserted. Both operations were performed as previously described [21]. After resection, reconstruction step involves fashioning pancreaticojejunostomy, hepaticojejunostomy, and gastrojejunostomy with a single proximal jejunoileal loop, with pancreaticojejunostomy performed first and hepaticojejunostomy fashioned immediately after with few centimeters in-between, then gastrojejunostomy fashioned on the same loop after 60 cm length. Specimen orientation to the pathologist was done. Important margins like the critical vessels’ margins were marked.

The operative time was calculated in minutes, starting from skin incision to skin closure. Blood loss was assessed in coordination with the anesthesia team by counting the number of soaked towels and gauze and calculating the amount of blood suctioned throughout the procedure in milliliters. The need for transfusion of blood products was assessed by the number of units transferred during or after the procedure.

### Postoperative care

All patients received routine nutrition regimen after PD. Amylase in the drain was measured when a postoperative pancreatic fistula (POPF) was suspected to confirm the diagnosis.

Daily chest assessment, serial abdominal examination, wound assessment, and pain intensity were recorded using the VAS at 6, 12, 24, and 48 h postoperatively. Postoperative pain was managed according to a standardized protocol for all patients. All participants received intravenous paracetamol (1 g every 8 h) and non-steroidal anti-inflammatory drugs unless contraindicated. Intravenous morphine (0.05–0.1 mg/kg) was administered as rescue analgesia for VAS scores exceeding 4. No local infiltration or regional blocks were used.

Further laboratory and radiological assessments were done if required. The patients received scheduled follow-up visits for 3 months, and morbidity according to Clavien–Dindo (CD) classification and 30-day mortality were recorded.

### Statistical methods

The statistical analysis was done using the IBM SPSS software package version 28.0 (IBM Corp, Armonk, NY, USA; 2021). Chi-square test, Fisher’s exact test, or *Z* test for proportion were used for the comparison of the categorical data as appropriate, while the numerical data were compared using the independent *t* test or Mann–Whitney test according to the data normality. A binary logistic regression test was used to assess predictors of mortality. The significance of the obtained results was judged at the 5% level.

## Results

### Patient enrollment and follow-up

**Fig. 1:**
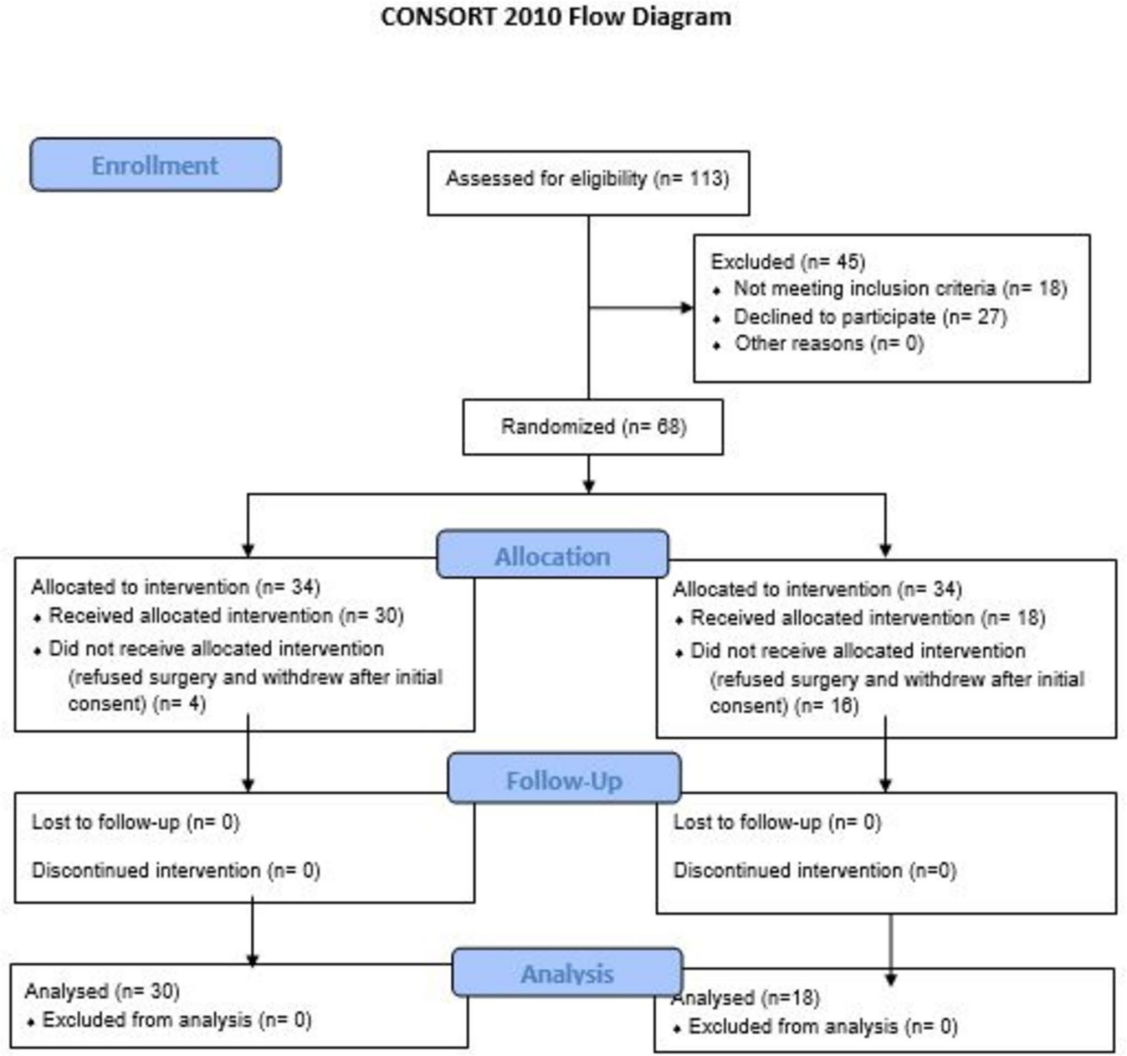
Patients flow chart

Patient recruitment was conducted between September 2022 and September 2023. All patients were followed for a period of 3 months postoperatively. The final follow-up was completed in December 2023. The trial was completed as planned after reaching the predefined sample size. There were no interim analyses or early stopping due to efficacy, futility, or safety concerns. Initially, the study included 68 patients who were eligible for the study and equally allocated to the two groups. Twenty patients discontinued the intervention, and finally, the study included 48 patients who underwent total laparoscopic (*n* = 30) or open (*n* = 18) PD for the surgical treatment of pancreatic and periampullary masses (Fig. 1). Reasons for post-randomization discontinuation in the study patients are shown in Table 1.

**Table 1:** Reason for post-randomization discontinuation in the study patients

Reason	LPD (*n* = 34)	OPD (*n* = 34)	Subsequent management
Patient/family refused major surgery a few days prior to the operation due to fear related to the patient’s frailty or advanced age	2	10	Removed from surgical schedule, offered non-operative palliative or supportive care as appropriate
Acute medical instability pre-induction (ventricular tachycardia storm, sepsis, or significant uncontrolled hypertension)	2	4	Surgery canceled; ICU/ward care; no PD within study window
Positive SARS-CoV-2 PCR on admission screen	–	2	Quarantined; PD re-scheduled after study period
Total discontinuations	4	16	

### Baseline characteristics

The mean age in the OPD group was 60.2 ± 9.1 years, and that of the LPD group was 54.1 ± 15.4 years (*p* = 0.130). The gender distribution revealed that the patients in both groups were predominantly males (*p* = 0.441) (Table 2).

**Table 2:** Baseline sociodemographic and clinical of the study patients

		LPD (*n* = 30)	OPD (*n* = 18)	*p* value
Age (years)				
Mean ± SD,		54.1 ± 15.4,	60.2 ± 9.1,	0.130^a^
		Count (%)	Count (%)	
Sex				
Female		10 (33.3%)	8 (44.4%)	0.441^b^
Male		20 (66.7%)	10 (55.6%)	
Comorbidities				
T2DM		6 (20.0%)	5 (27.8%)	0.535^c^
Hypertension		7 (23.3%)	5 (27.8%)	0.731^c^
HCV		2 (6.7%)	0 (0.0%)	0.273^c^
Lung fibrosis/COPD		1 (3.3%)	1 (5.6%)	0.709^c^
Bipolar depression		1 (3.3%)	0 (0.0%)	0.434^c^
Elevated CA19-9				
No		19 (63.3)	10 (55.6%)	0.594^b^
Yes		11 (36.7%)	8 (44.4%)	
Neoadjuvant therapy				
No		28 (93.3%)	15 (83.3%)	0.594^b^
Yes		2 (6.7%)	3 (16.7%)	
	Chemotherapy	2 (6.7%)	1 (5.6%)	0.878c
	Radiotherapy	0 (0.0%)	2 (11.1%)	0.062c

*T2DM* type 2 diabetes mellitus, *HCV* hepatitis C virus, *COPD* chronic obstructive pulmonary disease, a: t, t value of the student t test, b: *χ*^2^, Chi-square test, c: *Z* test for proportion

Elevated CA19-9 levels were observed in 36.7% of LPD patients and 44.4% of OPD patients (*p* = 0.594). Five patients received neoadjuvant therapy due to more extensive vascular involvement (Table 2). The imaging features including the diameter of the lesion, the diameter of pancreatic duct and CHD, and the vascular relations of the lesion were comparable in the two groups (Table 3).

**Table 3:** Preoperative imaging findings of the study patients

Mean ± SD		LPD (*n* = 30)	OPD (*n* = 18)	*p* value
Maximum diameter of the lesion (mm)		3.59 ± 1.83	4.03 ± 1.77	0.463^a^
Pancreatic duct diameter (mm)		5.72 ± 4	5.04 ± 2.7	0.582^a^
CHD diameter (mm)		12.5 ± 6.2	11.4 ± 5	0.594^a^
		Count (%)	Count (%)	
Relation to vessels				
Vascular involvement		7 (23.3%)	8 (44.4%)	0.127^b^
Vascular encasement		1 (3.3%)	2 (11.1%)	0.182^d^
Involved vessel	SMV	6 (20.0%)	8 (44.4%)	0.071^c^
	PV	1 (3.3%)	3 (16.7%)	0.106^c^
	GDA	1 (3.3%)	2 (11.1%)	0.709^c^

*CHD* common hepatic duct, *SMV* superior mesenteric vein, *PV* portal vein, *GDA* gastroduodenal artery, *RHA* right hepatic artery, a: t, t value of the student *t* test, b: *χ*^2^, Chi-square test, c: *Z*, *Z* test for proportion, d: Fisher’s exact test

### Intraoperative data

During surgery, soft pancreas was noted in 13.3% of laparoscopic cases compared to 33.3% in open cases (*p* = 0.099). Stent placement during surgery was done in 90% of the laparoscopic cases compared to 73.2% of the open cases (*p* = 0.11).

The mean operative time was 522 ± 130 min compared to 437 ± 154 min in open surgery, with a *p* value of 0.045. Intraoperative vascular injuries occurred in 2 patients in the LPD group (6.7%) (*p* = 0.273). Vascular resection was not required in laparoscopic cases (100.0%) compared to open cases (94.4%), with a *p* value of 0.192.

Blood loss during surgery was significantly lower in the LPD group (median of 300 ml) than in the OPD group (median of 500 ml) (*p* = 0.036). Similarly, the need for blood transfusion was 40.0% compared to 72.2% (*p* = 0.031). For cases requiring blood transfusion, the median number of units transfused was 2 in the two groups (*p* = 0.812).

### Postoperative outcomes

There was no statistically significant difference regarding the postoperative major complications. Postoperative complications summarized by highest Clavien–Dindo grade per patient in Table 4. The rates of clinically relevant postoperative pancreatic fistula (POPF, grade B/C) were comparable between the LPD and OPD groups (6.7% vs. 5.6%, *p* = 0.999). Postpancreatectomy hemorrhage (PPH) occurred in one patient (5.6%) in the OPD group and none in the LPD group (*p* = 0.375). Bile leak was observed in 3 patients (10.0%) following LPD and in 1 patient (5.6%) after OPD (*p* = 0.999). Conversion to open surgery or reoperation was required in 3 patients (10.0%) in the LPD group and none in the OPD group (*p* = 0.281). Delayed gastric emptying (DGE) occurred in 3 patients (10.0%) in the LPD group and 1 (5.6%) in the OPD group (*p* = 0.999).

**Table 4:** Postoperative complications summarized by highest Clavien–Dindo grade per patient

Complication type	LPD (n = 30)	OPD (n = 18)	p value
Overall complications	23 (76.7%)	14 (77.8%)	0.28
Clavien–Dindo grade			
Grade I–II	19 (63.3%)	8 (44.4%)	0.24
Grade III–IV	3 (10.0%)	4 (22.3%)	0.40
Grade V (mortality)	1 (3.3%)	2 (11.1%)	0.547
Specific surgical complications			
Clinically relevant POPF (B/C)	2 (6.7%)	1 (5.6%)	0.999
PPH	0 (0.0%)	1 (5.6%)	0.375
Bile leak	3 (10.0%)	1 (5.6%)	0.999
Conversion to open surgery/reoperation	3 (10.0%)	0 (0.0%)	0.282
Delayed gastric emptying (DGE)	3 (10.0%)	1 (5.6%)	0.999
Medical complications			
Pulmonary	3 (10.0%)	6 (33.3%)	0.063
Others	2 (6.7%)	3 (16.7%)	0.547
90-day mortality	1 (3.3%)	2 (11.1%)	0.27
Serious complications (≥ III)	4 (13.3%)	6 (33.3%)	0.08
Textbook Outcome (TO)	26 (86.7%)	11 (61.1%)	0.058
Failure to Rescue (FTR)	1/4 (25%)	2/6 (33.3%)	1.000
PAncreatic Surgery CE (PACE)	22 (73.3%)	13 (72.2%)	1.000

Textbook outcome (TO) was achieved in 86.7% of LPD cases compared with 61.1% of OPD cases (*p* = 0.058). Failure-to-rescue (FTR) was 25% in LPD and 33.3% in OPD (*p* = 1.000). The Pancreatic Surgery Complication Evaluation (PACE) score indicated similar rates between both groups (73.3% vs. 72.2%, *p* = 1.000).

Conversion to open surgery was indicated in 3 patients in the LPD group (10%). These were the two cases of intraoperative vascular injury and a third case where the pancreatic duct could not be identified during the laparoscopic approach. The LPD group showed a lower mortality rate (3.3%) than the OPD group (11.1%) (*p* = 0.281).

The duration of hospital stay was shorter in the LPD group (mean of 8.1 ± 5.6 days) than the OPD group (mean of 10.6 ± 6.1 days), with a* p* value of 0.049. Regarding ICU admission, the mean duration of the ICU stay was 1.7 ± 0.7 days for laparoscopic cases and 4.5 ± 7.0 days for open cases (p = 0.043).

There were no statistically significant differences regarding the duration to ambulating, time to GIT recovery, and the duration to food intake tolerance (Table 5).

**Table 5:** Postoperative functional recovery in the study patients

Mean ± SD	LPD (*n* = 30)	OPD (*n* = 18)	*p* value
Ambulation (days)	2.3 ± 1.8	2.7 ± 1.41	0.224^e^
Days to fluid intake tolerance	2.1 ± 0.8	2.9 ± 1.5	0.021^e^*
Days to food intake tolerance	3.6 ± 1	5.1 ± 3.5	0.106^e^
Return of bowel function (days)	2.8 ± 0.9	3.2 ± 1.2	0.256^e^
VAS score	4.4 ± 2.5	6.06 ± 2.0	0.022^e^*

*VAS* visual analog scale, e: Mann–Whitney test *: statistically significant

Assessment of pain scores showed that the LPD group had a mean score of 4.4 ± 2.5 to respective values of 6.1 ± 2.0 in the OPD group (*p* = 0.022) (Table 5).

### Histopathological findings

Histopathological examination demonstrated that the majority of cases in both LPD (80.0%) and OPD (94.4%) involved the pancreatic head (*p* = 0.345). The histological types of tumors varied among the cases, encompassing a range of pancreatic neoplasms. Pancreatic duct adenocarcinoma (PDAC) was the predominant type in both groups (53.3% and 77.8%, respectively) (*p* = 0.235).

Evaluation of margin status revealed that the majority of cases in both LPD (90.0%) and OPD (88.9%) had negative margins, indicating successful resection (*p* = 0.903). The mean number of dissected lymph nodes in LPD was 14.7 ± 8.3, and in OPD, it was 12.8 ± 6.1 (*p* = 0.539). The mean ratio of positive lymph nodes to dissected lymph nodes was higher in the LPD group (0.06 ± 0.1) than the OPD group (0.006 ± 0.02) (*p* = 0.011). The distribution of tumor (T) showed comparable results (*p* = 0.35), while N0, N1, and N2 constituted 53.3% (16/30), 40% (12/30), and 6.7% (2/30) compared to 88.9% (16/18), 11.1% (2/18), and 0% (0/18) in the OPD group, respectively (*p* = 0.038).

### Predictors of mortality

Univariable binary logistic regression analysis for potential predictors of mortality occurrence in patients who underwent PD surgery revealed that the absence of a stent was a significant predictor of mortality (*p* = 0.049). Hypertension was shown to be a marginally significant predictor of mortality (*p* = 0.076) (Table 6 in Supplementary File 1).

## Discussion

This study represents one of the few randomized controlled trials assessing the feasibility and safety of LPD compared to open pancreatoduodenectomy (OPD) [10, 22–25]. LPD in the present study was associated with significantly longer operative times compared to OPD. Intraoperative vascular injury occurred in two LPD cases, necessitating conversion to open surgery, while a third case required conversion due to difficulty identifying the pancreatic duct resulting in a conversion rate of 10%. It is expected that this rate will decrease as surgeons progress along the learning curve. Despite these challenges, blood loss in LPD cases was significantly lower than in OPD, with a median difference of 200 ml and a mean difference of 125 ml, translating into a reduced need for transfusion. The superior hemostatic control observed in the LPD group can be attributed to enhanced visualization through magnified, high-definition images, which allow meticulous tissue dissection and precise vascular control. The minimally invasive nature of LPD, with smaller incisions and reduced tissue trauma, contributes to decreased intraoperative blood loss, while the tamponade effect of pneumoperitoneum further enhances hemostasis.

In this study, LPD was associated with lower overall morbidity and major complication rates, though differences in major complications were not statistically significant. These outcomes corresponded with significantly shorter ICU and hospital stays, while mortality rates were comparable between LPD and OPD groups. Postoperative gastrointestinal recovery is a key target of enhanced recovery protocols. In the present study, LPD patients achieved earlier fluid and food tolerance and ambulation, likely due to reduced surgical trauma, lower pain, and milder stress response. Indeed, the LPD group in this study experienced significantly lower postoperative pain. Regarding oncologic outcomes, both groups demonstrated high R0 resection rates (90.0% for LPD and 88.9% for OPD) and similar numbers of retrieved lymph nodes, indicating effective tumor clearance. Finally, analysis of factors influencing mortality indicated that the absence of stent insertion was significantly associated with death. Stent placement aids pancreatic drainage, potentially reducing postoperative complications and pancreatic leakage, which are key contributors to morbidity and mortality. The present study identified hypertension as a marginally significant predictor of mortality.

Consistent with our findings, recent meta-analyses by Ausania et al., Lin et al., Nickel et al., and Yan et al. [12, 26–28] reported the significantly longer operative times in LPD compared to OPD. Our conversion rate aligns with previously reported rates ranging from 3.1% [27] to 24.1% [29] and is comparable to rates reported by Delitto et al., Chopinet et al., Chen et al., and El Nakeeb et al. [30–33] (9.1–10.8%).

The current study findings are comparable to previously reported differences of 200 ml by El Nakeeb et al. [33] and Dang et al. [34], 210 ml by Chen et al. [1], 197 ml by Ding et al. [35], and 151 ml by Palanivelu et al. [25]. Also, these study findings are corroborating the findings of Feng et al. [36], Sun et al. [37], and Aiolfi et al. [38] who demonstrated shorter hospital stays and fewer postoperative complications, De Rooij et al. [39] who reported faster gastrointestinal recovery after laparoscopic versus open PD, and Dang et al. [34], Van Hilst et al. [10], and Tang et al. [40] who reported the significantly lower pain scores associated with LPD.

The non-significant differences in R0 rates are supported by several studies [1, 24, 25, 29, 30, 41, 42], and Aiolfi et al. [38] reported similar lymph node retrieval and R0 rates between LPD and OPD, supporting the oncologic safety of LPD. The predictors of mortality in the current study are aligning with findings from the National Surgical Quality Improvement Program (NSQIP) Surgical Risk Calculator developed by the American College of Surgeons (ACS) in 2013 [43, 44] and other subsequent risk calculators designed to predict poor outcomes after pancreatoduodenectomy (PD) [45, 46]. In particular, Lin et al. [46], analyzing 14,806 adult PD patients, confirmed hypertension as a significant risk factor for adverse postoperative outcomes, likely due to its hemodynamic impact compounded by renal and cardiovascular comorbidities.

Several limitations should be noted. First, the study is a single-center trial with a modest sample size (30 LPD, 18 OPD), providing adequate statistical power only for the pre-specified primary endpoint (length of stay) and underpowered for infrequent but clinically important outcomes, such as grade IV complications, post-pancreatectomy hemorrhage, or mortality. Second, attrition after randomization—due primarily to late patient/family withdrawal and peri-induction instability—reduced the analyzable cohort and may have introduced residual selection bias. While the trial suggests that LPD offers short-term perioperative benefits over OPD, generalizability is limited by the single-center design, selective eligibility criteria, and the experience level of the surgical team.

Despite these limitations, this RCT provides valuable high-level evidence comparing LPD with OPD for pancreatic head and periampullary tumors. Although LPD was associated with a longer operative time (mean difference of 85 min), this difference is clinically modest given the complexity of PD and the learning curve associated with minimally invasive techniques. Operative time decreased as surgical proficiency increased, highlighting that initial time differences diminish with experience. More importantly, the slight prolongation of operative time is outweighed by the benefits of LPD, including reduced intraoperative blood loss, shorter postoperative recovery, and fewer postoperative complications, while maintaining comparable oncologic adequacy.

It is worthy to note that the adoption of minimally invasive and robotic-assisted PD (RPD) has been evidently varied across different regions, influenced by factors such as healthcare infrastructure, economic considerations, and training opportunities. In Europe and the United States, there has been a significant shift toward robotic-assisted PD. This trend is supported by the increasing availability of robotic platforms and a growing body of evidence favoring robotic approaches in complex surgeries. The EUROPA trial, a randomized controlled trial comparing RPD with OPD, is a pivotal study in this context [47]. Contrasting with the Western trend, many Asian countries continue to emphasize LPD. In Thailand, for instance, laparoscopic PD remains limited to a few centers due to its complexity and steep learning curve [48]. In Singapore, both laparoscopic and robotic pancreatic surgeries are performed, LPD being more prevalent [49].

In developing countries, the adoption of both LPD and RPD is hindered by several factors such as the high expense of robotic systems that limits their availability in many healthcare settings. Also, the steep learning curve associated with both LPD and RPD necessitates specialized training, which may not be readily accessible. In addition, adequate surgical infrastructure and support systems are often lacking, impeding the implementation of advanced surgical techniques.

## Conclusion

Despite a longer mean operative time associated with LPD, notably, LPD demonstrated advantages in terms of lower intraoperative blood loss, reduced need for blood transfusion, faster GIT recovery and ambulation, and a shorter hospital stay. The LPD oncological safety was evident in similar rates of negative margins and retrieval of lymph nodes when compared to OPD.

## Supplementary Information

Below is the link to the electronic supplementary material.

Supplementary file1 (DOCX 17 KB)
