# Supplementary material for: Laparoscopic versus open pancreatoduodenectomy: a pilot randomized trial in a developing African country
Source: Surg Endosc. 2025 Dec 4;40(2):1641–50. doi: 10.1007/s00464-025-12377-x (PMC12881053; doi:10.1007/s00464-025-12377-x)
Supplement: Supplementary file 1 — Supplementary file1 (DOCX 17 KB) [file 464_2025_12377_MOESM1_ESM.docx]

**Table 6: Univariable binary logistic regression analysis for the predictors of mortality**

| **Variable** | **p-value** | **OR** | **95% CI** |
| --- | --- | --- | --- |
| **Age** | 0.539 | 1.04 | [0.926, 1.16] |
| **Sex** | 0.878 | 1.21 | [1.0, 14.4] |
| **BMI Obese** | 0.246 | 1.7 | [0.332, 8.67] |
| **Overweight** | 0.997 | 2.11*10^8^ | [0.0, inf] |
| **Underweight** | 0.998 | 2.11*10^8^ | [0.0, inf] |
| **T2DM** | 0.996 | 3.61*10-^8^ | [0.0, inf] |
| **Hypertension** | 0.076 | 4.47 | [0.856, 23.37] |
| **HCV** | 0.996 | 3.37*10^-7^ | [0.0, inf] |
| **Lung fibrosis/COPD** | 0.996 | 3.37*10^-7^ | [0.0, inf] |
| **Bipolar depression** | 0.995 | 9.37*10^-7^ | [0.0, inf] |
| **Abdominal pain** | 0.996 | 3.5*10^-8^ | [0.0, inf] |
| **Anorexia** | 0.997 | 1.21*10^-7^ | [0.0, inf] |
| **Vomiting** | 0.996 | 3.37*10^-7^ | [0.0, inf] |
| **Weight loss** | 0.996 | 1.18*10^-7^ | [0.0, inf] |
| **Melena** | 0.995 | 9.37*10^-7^ | [0.0, inf] |
| **Obstructive jaundice** | 0.308 | 0.276 | [0.232, 3.28] |
| **Cholangitis** | 0.995 | 9.37*10^-7^ | [0.0, inf] |
| **Maximum diameter** | 0.243 | 1.8 | [0.67, 4.86] |
| **Pancreatic duct diameter** | 0.105 | 1.33 | [0.942, 1.88] |
| **CHD diameter** | 0.732 | 1.05 | [0.812, 1.35] |
| **Vascular involvement** | 0.995 | 3.18*10^-8^ | [0.0, inf] |
| **Vascular encasement** | 0.997 | 1.21*10^-7^ | [0.0, inf] |
| **Anatomical variance** | 0.995 | 1.10*10^-7^ | [0.0, inf] |
| **Elevated CA19-9** | 0.996 | 1.01*10^-8^ | [0.0, inf] |
| **Neoadjuvant therapy** | 0.996 | 1.15*10^-7^ | [0.0, inf] |
| **Surgical approach** | 0.308 | 3.63 | [0.305, 43.15] |
| **Soft pancreas** | 0.342 | 0.732 | [0.128, 18.7] |
| **Pylorus preserving** | 0.820 | 0.750 | [0.06, 8.9] |
| **Stent placement** | 0.049* | 0.08 | [0.006, 0.985] |
| **Operative time** | 0.458 | 0.779 | [0.988, 1.01] |
| **Intraoperative vascular injury** | 0.996 | 3.92*10^-8^ | [0.0, inf] |
| **Blood loss** | 0.554 | 0.999 | [0.995, 1.003] |
| **T** | 0.658 | 0.730 | [0.18, 2.94] |
| **N** | 0.897 | 1.13 | [0.17, 7.72] |
